# Segmented relations between online reading behaviors, text properties, and reader–text interactions: An eye-movement experiment

**DOI:** 10.3389/fpsyg.2022.1006662

**Published:** 2023-01-11

**Authors:** Tao Gong, Lan Shuai

**Affiliations:** ^1^Haskins Laboratories, New Haven, CT, United States; ^2^School of Foreign Languages, Zhejiang University of Finance and Economics, Hangzhou, Zhejiang, China; ^3^Educational Testing Service, Princeton, NJ, United States; ^4^Google, New York, NY, United States

**Keywords:** eye-movement, lexical properties, individual differences, mixed-effects regression, segmented linear regression

## Abstract

**Purpose:**

To investigate relations between abilities of readers and properties of words during online sentence reading, we conducted a sentence reading eye-movements study on young adults of English monolinguals from the US, who exhibited a wide scope of individual differences in standard measures of language and literacy skills.

**Method:**

We adopted mixed-effects regression models of gaze measures of early and late print processing stages from sentence onset to investigate possible associations between gaze measures, text properties, and skill measures. We also applied segmented linear regressions to detect the dynamics of identified associations.

**Results:**

Our study reported significant associations between (a) gaze measures (first-pass reading time, total reading times, and first-pass regression probability) and (b) interactions of lexical properties (word length or position) and skill measures (vocabulary, oral reading fluency, decoding, and verbal working memory), and confirmed a segmented linear dynamics between gaze measures and lexical properties, which was influenced by skill measures.

**Conclusion:**

This study extends the previous work on predictive effects of individual language and literacy skills on online reading behavior, enriches the existing methodology exploring the dynamics of associations between lexical properties and eye-movement measures, and stimulates future work investigating factors that shape such dynamics.

## 1. Introduction

Contemporary views of reading highlight connections among cognitive abilities of readers, properties of texts, reading comprehension, and online reading behavior. The simple view of reading (SVR) proposes that reading comprehension is a function of visual word recognition, decoding, and language comprehension, the first two of which are print-specific aspects of reading skill (Gough and Tunmer, 1986), and the latter is construed as an *amodal* (not limited to a particular module like reading or listening) aspect of language. However, how language and literacy skills relate to lexical properties (e.g., word frequency, length, predictability, and position in sentence) and online reading behavior remains *implied*, at best, in SVR. In addition, the self-teaching hypothesis (STH) (Share, 1995) proposes that decoding allows developing readers to transform unfamiliar printed letter strings into recognizable sounds from their spoken language. This process helps readers to internalize the orthographic features of new words. Although highlighting that decoding skill predicts development of reading comprehension, thus being necessary for a reader to learn all words, orthographically regular or not, STH does not state explicitly how decoding helps comprehension during online sentence reading. Furthermore, the lexical quality hypothesis (LQH) (Perfetti, 2007) and the verbal efficiency theory (VET) (Perfetti, 1985) advocate that what distinguishes good and poor readers is the ability to efficiently map orthographic forms to phonological representations, and ultimately to semantics. However, it is unclear how different aspects or levels of language and literacy skills influence reading processes.

Existing studies of individual differences in reading often focus on offline outcomes (e.g., reading comprehension), and these outcomes are in fact cumulative end products of various processes involved in meaning construction (Snow, 2002). Recent studies have begun to shift their attentions from reading outcomes to reading processes, as in moment-to-moment measures (e.g., eye-movements) of reading behavior (e.g., Traxler, 2007; Rayner, 2009b; Kuperman and Van Dyke, 2011; Radach and Kennedy, 2012; Rayner et al., 2012, 2015; Kuperman et al., 2018). Eye-movement patterns during reading are found to vary with lexical properties (Rayner and Duffy, 1986; Rayner, 1998; Joseph et al., 2013). In addition, eye-movement patterns also rely on cognitive capacities that support reading. The dynamics of information processing during reading is governed not only by lexical properties of the text (Radach and Kennedy, 2012), but also by knowledge and cognitive resources of the reader (Beck et al., 1982; Gough and Tunmer, 1986; Hoover and Gough, 1990; Catts et al., 2006). Reading comprehension emerges as a juxtaposition of the lexical properties and the skills, knowledge, and experience of the reader (Perfetti and Lesgold, 1977; Nelson Taylor and Perfetti, 2016; Kuperman et al., 2018).

Many previous studies have reported the “direct” roles of language and literacy skills in reading outcomes or predicting online reading behavior, but there lack enough investigations on whether those skills could also “indirectly” influence reading behavior through interactions with lexical properties, given that lexical properties are central to regulation of gaze behavior during connected text reading and influence of effortful lexical, syntactic, semantic, and pragmatic processing (Duffy et al., 1988; Rayner, 2009a). In addition, many studies have focused selectively on university students (there are exceptions though, e.g., one on participants of similar age and skill to those in our study (Kuperman and Van Dyke, 2011), and two on readers younger than those in our study (Joseph et al., 2013; Valle et al., 2013). University students often have a narrow range of language and literacy skills centered above average, which makes them insufficient to reveal the potentially much wider scope of individual differences in those skills and the general effects of such differences on online reading behavior (Henrich et al., 2010). Furthermore, through simple regression analyses, many existing studies only reported whether or not an online reading behavior is correlated with certain lexical properties and/or individual skills, yet there lack investigations on the dynamics of identified relations, e.g., does an identified correlation follow a simple linear relation, a nonlinear relation, or else? Given that there has been accumulated evidence informing us about what lexical properties or individual skills may or may not influence or be correlated with online reading behavior, it is time to further examine the dynamics of such causal or correlational relations concerning lexical properties, individual skills, and online reading behavior.

Noting these and given the dearth of research on how differences in basic reading skills and vocabulary exert influence on online reading at a sentence level, this study was designed to yield empirical data and inform relevant theories. Based on eye-movement measures and online reading process, this study aimed to investigate two research questions:

(a) Can interactions between language and literacy skills and lexical properties influence online reading behavior?
(b) What is the dynamics of the correlation between online reading behavior and lexical properties?

Answers to these questions will bring an intimate view of reading process at the levels of words, phrases, and larger units, and contribute to the research on how lexical properties and individual differences in language and literacy skills jointly affect online reading behavior.

Following a data-driven approach, this study centered on the skills concerned with reading process as gauged by online reading behaviors, and investigated how these skills interact with lexical properties during online reading. In view of the existing theories (e.g., SVR, STH, LQH, and VET), we focused on four skills: decoding, reading fluency, word knowledge (vocabulary), and working memory (see next section for details). Some of them were omitted in early studies [e.g., working memory was not included by Kuperman and Van Dyke (2011)]. In our study, all these skills were assessed by a battery of standard tests. In addition, as part of a general research program aimed at developing profiles of adolescent and young adult readers (aged from 16 to 25 years), especially those whose educational and occupational prospects might be constrained by their limited language and literacy skills, our study targeted on non-university students, who possess a much wider range of individual differences in these skills than would typically be found in university students (Braze et al., 2007). This enables a more detailed examination of the role of individual differences in reading behavior than would be possible with a more restricted range of differences (Peterson, 2001). Furthermore, after identifying significant interactions between lexical properties and individual skills on online reading behavior gauged by eye-movement measures, we further quantified the dynamics between the aspects involved in the significant interactions, i.e., the lexical properties and the eye-movement measures in participants with high and low levels of the skills. This data-driven analysis helps reveal how the skills influence online reading behavior via interactions with lexical properties.

In terms of methodology, we applied mixed-effects regression models on rich observations, and carefully controlled the family-wise errors, collinearity, and overfitting. This type of models can simultaneously address the main effects of the skill measures and their interactions with lexical properties in one model, and collectively reveal the key interactions with lexical properties. In addition, we designed a way to visualize the correlations between eye-movement measures and lexical properties under different levels of skills, and selected among popular regression models the “best” one to reflect the dynamics. A similar method was practiced to detect quantitative relations between decoding skills and comprehension scores in reading assessment (Wang et al., 2019).

Our study did not find significant effects attributable to individual skill differences, due primarily to the wider spans of the abilities in our participants than those of university students recruited in previous studies. Nonetheless, we identified significant interactions between lexical properties and individual skills, including: interactions between word length and verbal working memory and oral comprehension plus vocabulary in regulating first-pass reading time, interactions between word position and oral reading fluency and verbal working memory in shaping total reading times, and interaction between word position and decoding in adjusting first-pass regression probability. Our analysis revealed a segmented linear dynamics between lexical properties and eye-movement measures, which could be further manipulated by individual skills. All these findings reveal important predictability of those skills on online reading behavior, and contribute to theoretical discussions on how those skills regulate reading behavior at a sentence level through interactions with lexical properties. Note that more research is needed to better understand what factors shape the pivot points in the segmented linear curves.

## 2. Target skills and recent studies on them

Among various language and literacy skills, we focused on four of them.

*Decoding* is the ability to apply the orthography-to-phonology correspondence rules to pronounce written words. It is essential to translating print to spoken language, and includes, at least, the knowledge of letter patterns and letter-sound relationships, upon which all other reading skills are built (Share, 1995). SVH claims that decoding, together with listening comprehension, makes substantial contributions to variation in reading comprehension. Studies have revealed that reading comprehension differences are associated with decoding skill differences in children and adolescent readers (Shankweiler et al., 1999) and that the ability to retrieve phonological cues can predict individual differences in reading fluency (Barth et al., 2009). Studies of online reading processes have discovered that a high decoding skill enables a rapid access to a word’s orthographic form and its meaning, thus accelerating word naming speed (Manis and Freedman, 2001) and reflecting high text-level reading fluency and word-level recognition during connected text reading (Wolf et al., 2002).

*Reading fluency* is the ability to read connected text quickly, accurately, and with expression. Conventional measures like the Gray Oral Reading Test (Wiederholt and Bryant, 2001) assess oral reading fluency. Recent tests measure this skill through silent reading, e.g., the Silent Reading Efficiency and Comprehension Test (Wagner et al., 2010). Regardless of modality, reading fluency measures draw on important capacities to lexical access (Perfetti, 1985) and mediate reading comprehension (e.g., Tilstra et al., 2009; Macaruso and Shankweiler, 2010; Silverman et al., 2012). Longitudinal and corpus-based studies have shown that reading fluency is a reliable index of reading comprehension in students (Fuchs et al., 2001; Miller and Schwanenflugel, 2008; Reschly et al., 2009; Petscher and Kim, 2011) and it performs as well as or better than other reading comprehension tests as a predictor for higher stakes comprehension tasks (Baker et al., 2008; Marcotte and Hintze, 2009). Eye-movement studies have also revealed that phonemic awareness, a known predictor for early word recognition and decoding, contributes to reading fluency (Ashby et al., 2013).

*Vocabulary* is another key component of reading skills. Orally assessed vocabulary knowledge captures variance in reading comprehension, even if comprehension and decoding skill are accounted for (Braze et al., 2007; Tunmer and Chapman, 2012). Vocabulary breadth and depth, as well as semantic relatedness can predict individual differences in reading comprehension of fourth-grade students (Swart et al., 2016). Oral vocabulary makes an independent contribution to reading comprehension in grade school children (Ouellette and Beers, 2010) and young adult readers (Braze et al., 2007), and serves as a strong predictor for reading comprehension in typically developing Grades 1–3 students and dyslexic readers of Grades 4–5 (Chik et al., 2010). During sentence reading, high-vocabulary readers are found more likely to make online elaborative inferences than low-vocabulary ones (Calvo et al., 2003). Nelson Taylor and Perfetti (2016) report that: readers with greater knowledge of less common words tend to read faster and with greater accuracy in paragraph reading, and the amount of exposure to phonological and semantic constituents of words during training modulates re-reading behavior in this process.

*Verbal working memory* enables readers to hold on to verbal cues to comprehend lengthy or complex sentences, and thus facilitates readers’ abilities to derive compositional meanings of sentences. High working memory capacity can accelerate the time course of predictive inferences during sentence reading (Estevez and Calvo, 2000). Compared to readers with higher working memory capacity, those with lower capacity exhibit more difficulties (in terms of longer regression and total fixation time) in associating relative clauses with preceding fragments (Traxler, 2007), and spend more time re-reading ambiguous regions of texts (Clifton et al., 2003). Higher working memory capacity is also associated with higher reading fluency (with lower gaze durations and fewer look-backs from the final word of a sentence) (Calvo, 2004).

Motivated by previous studies on those skills, our study attempted to explore how reader-text interaction predicts reading patterns between good and poor readers differing in those skills.

In this line of research, existing studies often focus on identifying (by mixed-effects or generalized regression models, or machine learning models) the language and literacy skills that directly or indirectly (via interaction with lexical properties) cast important effects on reading process, but rarely touch upon the *dynamics* of any identified correlations between lexical or individual properties and reading process, e.g., whether and how the correlation between target skills and lexical properties change alongside the levels of the skills. For example, a recent study (Kuperman and Van Dyke, 2011) has shown that individual scores in rapid automatized letter naming (RAN) and word identification tests can supersede the effects of word length and frequency at early processing stages, and serve as stronger predictors than word frequency across eye-movement measures. However, family-wise Type-I error was not carefully controlled in the analyses (e.g., the same critical *p* value of 0.05 was used over 150 models involving multiple predictors that are correlated with each other), which weakens the claims that those skill measures are reliable predictors for online reading behavior.

Another study from the same group (Kuperman et al., 2018) incorporated more cognitive and linguistic skills, used sentence stimuli with increasing lexical, syntactic, and discourse complexity, and adopted random forest models to detect key predictors for eye-movement measures. This work analyzed the effects of lexical properties, individual skills, interactions between word length and those skills, and sentence complexity on eye-movements around words inside sentences, at the end of sentences, and whole passages. The analyses reported reading habit, vocabulary size, reading efficiency, vocabulary IQ, and rapid naming scores as key predictors on eye-movement patterns during online reading.

This data-driven approach fails to identify multiple factors having dominant and comparatively small yet still important effects. In a random forest model, extremely-high relative importance score of a predictor could mask the roles of other predictors. Since importance scores are relative to predictors, one random forest model cannot address all possible interactions between lexical properties and skill measures. In addition, the work *indirectly* examined the effects of interactions with word length: word length was segmented into long and short groups, and two random forest models were fitted respectively on the two groups to detect important skill measures whose effects exhibited different tendencies between the two models. The arbitrary, binary segmentation of word length groups presumes that if a skill measure has an influence on word length, the tendencies of the effect should be different on short and long words. This is not always the case; some factors may take effect on very long words, and others may trigger different reading patterns on very short words. A question on whether reading processes differ between individuals with high and low levels of skills is more meaningful than whether such processes differ between long and short words; in this sense, segmentation on skill levels is more informative than segmentation on lexical properties.

## 3. Materials and methods

The data in this study consisted of: (a) participants’ skill measure data obtained from a battery of standard psycho-educational tests; and (b) their eye-movement data gathered in a sentence reading experiment. The data were collected by trained research assistants. Informed consent was obtained from the participants of at least 18 years old; for those under 18, the participants provided assent and their parents or guardians signed written permissions. All participants were paid a proper remuneration for completing the protocols reported here together with the fMRI protocols reported elsewhere (Shankweiler et al., 2008; Braze et al., 2011). The procedures described here took ∼3.5 h; breaks were provided as needed.

### 3.1. Participants

Forty-five participants (age in 16–25 years, 27 females) were recruited from adult education centers, community college, and neighborhood-gathering places. Some participants had their secondary schooling interrupted but were then seeking a high school equivalency certificate or resuming work toward a regular high school diploma. At the time of experiment, most participants were enrolled in education programs (e.g., high school, adult school, or community college) (Braze et al., 2007, 2016). All participants were English monolinguals, and had normal or corrected-to-normal vision. They were prescreened to ensure the ability of reading simple sentences with comprehension. Data from one participant were excluded due to not completing all study components.

According to the power analysis in mixed-effects models (Brysbaert and Stevens, 2018), this number of sample size, together with the rich amount of eye-movement observations obtained during reading of multiple (72) sentences containing numerous (358) word types (see section 3.3 Materials and design), is sufficient to detect reliable significant factors.

### 3.2. Skill measures

Each participant was assessed in six domains of language and literacy skills, which served as the bases for analysis. Table 1 shows the raw (and normative wherever available) scores of each measure and a key to the labels of them. The domains and the tests used to measure them were:

**TABLE 1:** Raw scores and keys of the skill measures over 44 participants.

Name	Label	Mean	SD	Min.	25%	50%	75%	Max.	Skew	Kurtosis	Lambda
Age	–	20.61	2.27	16.6	18.73	20.16	22.41	25.49	0.30	-0.96	
Vocabulary	ppvt	172.41	17.64	132.00	160.50	176.50	187.00	196.00	-0.60	-0.71	1.18
	std.-score	103.39	14.65	78.00	92.00	102.00	115.00	132.00	0.12	-0.99	
	wasi.v	57.36	8.33	39.00	49.00	57.50	62.50	76.00	0.22	-0.73	
	t-score	53.25	9.73	36.00	44.00	52.50	60.00	74.00	0.38	-0.77	
Listening comprehension	piat.l	93.95	6.16	76.00	92.00	96.00	98.50	100.00	-1.34	0.95	2.08
	grade equiv.	12.00	1.84	6.90	11.60	13.00	13.00	13.00	-1.78	1.82	3.50
Decoding	wid	67.61	5.38	56.00	63.00	68.00	72.00	76.00	-0.19	-1.12	
	grade equiv.	13.18	4.81	5.60	8.50	12.70	19.00	19.00	0.05	-1.62	
	watt	27.23	3.06	20.00	25.50	28.00	30.00	32.00	-0.55	-0.66	1.38
	grade equiv.	10.83	4.67	4.30	7.10	10.20	15.40	19.00	0.40	-1.17	
Reading Comprehension	piat.r	89.48	10.25	68.00	83.50	95.00	97.50	99.00	-0.92	-0.57	1.34
	grade equiv.	10.79	2.86	5.00	8.50	13.00	13.00	13.00	-0.83	-0.92	1.62
	gort.comp	11.75	2.47	4.00	10.00	12.00	14.00	15.00	-0.68	0.38	1.57
Oral Reading Fluency	gort.wpm	177.01	39.04	87.34	149.91	176.82	197.81	288.80	0.39	0.31	
Verbal Working Memory	sspan.corr	31.88	5.78	20.00	27.00	33.00	36.50	42.00	-0.30	-1.04	

“Lambda” is for Box-Cox transformation for highly skewed scores; ppvt, Peabody Picture Vocabulary Test-Revised; wasi.v, Wechsler Abbreviated Scales of Intelligence Expressive Vocabulary Test; wid, Woodcock-Johnson-III Word Identification subtest; piat.l, even-numbered items in the Reading Comprehension subtest of the Peabody Individual Achievement Test-Revised; watt, Woodcock-Johnson-III Test of Achievement Word Attack subtest; piat.r, Peabody Individual Achievement Test-Revised; gort.comp, Gray Oral Reading Tests; gort.wpm, reading speed (words per minute) for Passages 5, 7, and 9 from the Gray Oral Reading Test; sspan.corr, listening version of the Sentence Span task.

(1) *Vocabulary*, assessed by the Peabody Picture Vocabulary Test-Revised (ppvt) (Dunn and Dunn, 1997) and the Wechsler Abbreviated Scales of Intelligence Expressive Vocabulary Test (wasi.v) (Psychological Corporation, 1999). Table 1 shows both raw and standard scores (normative sample mean = 100, SD = 15) of ppvt and both raw and *t*-scores (normative sample mean = 50, SD = 10) of wasi.v. Differences in word knowledge stem from (a) variations in language experience (in speech or print) and (b) differences in the ability to profit from it. Vocabulary is a good proxy for general, amodal, language ability of the community sample recruited in our study (Braze et al., 2016).
(2) *Listening comprehension*, assessed by the even-numbered items from the Reading Comprehension subtest of the Peabody Individual Achievement Test-Revised (piat.l) (Markwardt, 1998). Using the odd numbered items from this test for reading comprehension and the even numbered items for listening comprehension gives us a pair of tests well matched in task demand for both input modalities. Table 1 shows both raw and grade equivalent scores, the latter of which were calculated following Markwardt (1998) (see Braze et al., 2007 for details). Knowledge of vocabulary, compositional semantics, and syntax constitute the bases of oral language comprehension (Birch and Rayner, 1997; Frisson and McElree, 2008). The ability to understand language presented to the ear is a good indicator of general, amodal, language comprehension ability.
(3) *Decoding*, assessed by the Woodcock-Johnson-III Word Identification subtest (wid) (Woodcock et al., 2001) and the Woodcock-Johnson-III Test of Achievement Word Attack subtest (watt) (Woodcock et al., 2001). These are untimed tests for the ability to accurately pronounce printed words and non-words. Table 1 contains both raw and grade equivalent scores of the two measures.
(4) *Reading comprehension*, assessed by the odd numbered items from the Reading Comprehension subtest of the Peabody Individual Achievement Test-Revised (piat.r) (Markwardt, 1998) and the accuracies of the Passages 5, 7, and 9 from the Gray Oral Reading Test (gort.comp) (Wiederholt and Bryant, 2001). Calculation of grade equivalent scores of piat.r followed Braze et al. (2007). There were no standard scores of gort.comp, due to using only a subset of passages. Reading comprehension has been usefully thought of as the product of an individual’s facility with language and decoding skill (Gough and Tunmer, 1986).
(5) *Oral reading fluency*, assessed as the reading speed (words per minute) for Passages 5, 7, and 9 from the Gray Oral Reading Test (gort.wpm); the total number of words in these passages is 361 (Wiederholt and Bryant, 2001). There were no standard scores, since the measure was based on an abbreviated form of the Gray Oral Reading Test. Oral reading fluency consists of visual scanning, decoding, and high level language processing (Silverman et al., 2012).
(6) *Verbal working memory*, assessed by a listening version of the Sentence Span task (sspan.corr) (Daneman and Carpenter, 1980). This ensures non-confoundness with reading skills. Verbal working memory has been shown to account for differences in vocabulary growth independent of language exposure (Gathercole and Baddeley, 1989; Gathercole et al., 1999; Gupta, 2006).

These individual difference measures can be grouped into two sets: those explicitly linked to reading ability (reading comprehension, decoding skill, and oral reading fluency), and those not (listening comprehension, vocabulary, and verbal working memory) (Gough and Tunmer, 1986; Hoover and Gough, 1990). They tap into abilities equally important to comprehension, no matter whether the language input arrives by ear or by eye.

In addition to these domains, we also assessed *print experience* by a magazine title recognition checklist (MRT) and an author recognition checklist (ART) (cf., Stanovich and Cunningham, 1992) to gauge a person’s experience with language in printed form, which for literate individuals may well be a substantial part of their overall language experience, and *visual working memory* based on a computerized version of the Corsi Blocks task (corsi) (Corkin, 1974) implemented in Psyscope (Cohen et al., 1993). Given the fact ART and MRT only show high validity and reliability in proficient readers (e.g., university students) in their dominant language (McCarron and Kuperman, 2021), whereas our study is based upon participants having a wide span of reading skills, we excluded print experience in the regression analyses. In addition, compared to visual working memory, verbal working memory is more relevant to our sentence reading experiment, so we also excluded visual working memory in the regression analyses.

Prior to regression modeling, we examined the distributions of raw scores for deviations from normality. Several scores showed high skewness (absolute values over .5). To them, we applied Box-Cox transformations (Box and Cox, 1964) using the bcpower function in the R package *car* (Fox and Weisberg, 2011). All variables, transformed or not, were standardized by converting to *Z*-scores. Table 2 is the correlation table of the transformed and standardized measures (cf., Braze et al., 2007, 2016).

**TABLE 2:** Correlations between the age and the 9 skill measures, after Box-Cox transformation (for ppvt, watt, piat.r, and gort.comp) and standardization.

Measures	1	2	3	4	5	6	7	8	9
1. Age									
2. ppvt	0.591								
3. wasi.v	0.378	0.829							
4. piat.l	0.408	0.638	0.541						
5. wid	0.487	0.816	0.716	0.441					
6. watt	0.075	0.376	0.354	-0.025	0.613				
7. piat.r	0.550	0.798	0.718	0.634	0.714	0.317			
8. gort.comp	0.351	0.673	0.610	0.625	0.586	0.367	0.648		
9. gort.wpm	0.374	0.577	0.577	0.177	0.617	0.347	0.481	0.348	
10. sspan.corr	0.319	0.626	0.669	0.380	0.601	0.392	0.573	0.557	0.474

*n* = 44, | *r*| ≥ 0.24 corresponds to *p* < 0.05; | *r*| ≥ 0.31 to *p* < 0.01; | *r*| ≥ 0.39 to *p* < 0.001. ppvt, Peabody Picture Vocabulary Test-Revised; wasi.v, Wechsler Abbreviated Scales of Intelligence Expressive Vocabulary Test; piat.l, even-numbered items in the Reading Comprehension subtest of the Peabody Individual Achievement Test-Revised; wid, Woodcock-Johnson-III Word Identification subtest; watt, Woodcock-Johnson-III Test of Achievement Word Attack subtest; piat.r, Peabody Individual Achievement Test-Revised; gort.comp, Gray Oral Reading Tests; gort.wpm, reading speed (words per minute) for Passages 5, 7, and 9 from the Gray Oral Reading Test; sspan.corr, listening version of the Sentence Span task.

To reduce collinearity and the total number of predictors in the regression models, we combined measures tapping into common latent constructs. This was done by (a) taking the average of the transformed and standardized scores, and then (b) converting the average scores back to *Z*-scores. Measures of vocabulary and listening comprehension were combined into a composite measure of oral comprehension plus vocabulary (*oral.comp*) (Tunmer and Chapman, 2012; Braze et al., 2016; Kukona et al., 2016). Composites were also derived for decoding (*decod.comp*) and reading comprehension (*readcomp.comp*). Table 3 shows the correlation table of the centered and transformed skill measures.

**TABLE 3:** Correlations between the age and the 5 composite or independent measures.

Measures	1	2	3	4	5
1. Age					
2. oral.comp	0.520				
3. decod.comp	0.313	0.231			
4. readcomp.comp	0.497	0.844	0.608		
5. gort.wpm	0.374	0.503	0.536	0.457	
6. sspan.corr	0.319	0.632	0.553	0.622	0.474

*n* = 44, | *r*| ≥ 0.24 corresponds to *p* < 0.05; | *r*| ≥ 0.31 to *p* < 0.01; | *r*| ≥ 0.39 to *p* < 0.001. oral.comp, oral comprehension plus vocabulary, a composite variable of ppvt (Peabody Picture Vocabulary Test-Revised), wasi.v (Wechsler Abbreviated Scales of Intelligence Expressive Vocabulary Test) and piat.l (even-numbered items in the Reading Comprehension subtest of the Peabody Individual Achievement Test-Revised); decod.comp, decoding skill, a composite variable of wid (Woodcock-Johnson-III Word Identification subtest) and watt (Woodcock-Johnson-III Test of Achievement Word Attack subtest); readcomp.comp, reading comprehension skill, a composite variable of piat.r (Peabody Individual Achievement Test-Revised) and gort.comp (Gray Oral Reading Tests); gort.wpm, reading speed (words per minute) for Passages 5, 7, 9 from the Gray Oral Reading Test; sspan.corr, listening version of the Sentence Span task.

It is not surprising that the correlation between reading comprehension and oral comprehension plus vocabulary is high, since oral knowledge is an important indicator of reading comprehension (see section 2. Target skills and recent studies on them). Table 4 shows the statistics of the regression models between reading comprehension and oral comprehension plus vocabulary, decoding, and both, respectively. Consistent with early findings (Braze et al., 2007), a combination of both skills largely explains the variation of reading comprehension: *R*^2^ of the model using decoding is .370, *R*^2^ of the model using oral comprehension plus vocabulary is .712, and multiple *R*^2^ of the regression model using both decoding and oral comprehension plus vocabulary is .738. Notably, we exclude reading comprehension from the list of predictors in the regression models.

**TABLE 4:** Regression models targeting reading comprehension.

Model A:	Est.	SE	*t*	*p*	*R* ^2^
Decoding	0.608	0.123	4.964	0.00001	0.370
**Model B:**					
Oral comprehension plus vocabulary	0.844	0.083	10.190	<0.00001	0.712
**Model C:**					
Decoding	0.195	0.097	2.016	0.0503	0.738
Oral comprehension plus vocabulary	0.743	0.097	7.593	<0.00001	

Model A: Using decoding to predict reading comprehension; Model B: Using oral comprehension plus vocabulary to predict reading comprehension; Model C: Using both decoding and oral comprehension plus vocabulary to predict reading comprehension. *R*^2^ is the proportion of variance captured by a given variable after considering all other predictors in the model.

After these preprocessing stapes, the skill measures used in our regression analyses are: (a) oral comprehension plus vocabulary (oral.comp); (b) decoding (decod.comp); (c) oral reading fluency (gort.wpm); and (d) verbal working memory (sspan.corr).

### 3.3. Materials and design

Participants were asked to read 72 individual sentences while their eye-movements were recorded. Presentation order was pseudo-random across participants. These sentences were filler items in a study of comprehension process in young adults with limited literacy skills (Braze et al., 2006). All of the sentences were grammatical and transparent in meaning. The word types in them were carefully selected among high frequent words, and common names for persons, states, or holidays. The linguistic aspects of these sentences, such as part of speech or syntactic complexity, were carefully controlled. Supplementary Table 1 shows the complete list of the sentences. Many of these sentences were simple in terms of structure; forty-six stimuli sentences (over 79%) had no embedding structures, e.g., “Most of the students will be going to the class picnic next month.”; and the other 26 had one dependent clause, e.g., “The waiter had told the customer that the pies were fresh.” There were 503 unique word types (819 word tokens) in these sentences, an average of 11.375 word tokens per sentence (range = 11–16). Note that previous studies on university students involved sentences with increasing complexity in semantics and syntax (e.g., Kuperman and Van Dyke, 2011; Kuperman et al., 2018)), we leave the investigation of the relations between sentence complexity, reading skills, and online reading behavior for future work.

Before the experiment, we asked some individuals to evaluate the understandability of these sentences, based on a scale of 5, from “easy to understand” to “hard understand”. These individuals were recruited similarly as the experiment participants, but did not participate the experiment. All of them marked the filler sentences as “easy to understand”.

For each word in a sentence, we recorded its ordinal position in the sentence (note that the sentence initial and final words were excluded), its length in characters (Len_*W*_), and its frequency of occurrence per million words (Freq_*W*_). Word position is a context-dependent property, but word length and frequency are independent of sentence. Lexical frequencies were obtained from the Corpus of Contemporary American English (COCA).^1^ Frequency summaries for our materials exclude contractions (*n* = 2) and proper nouns (*n* = 23), both having no COCA frequencies. Possessive forms (*n* = 6) used the COCA frequencies of their uninflected forms. Analyses otherwise included all the remaining words found in the sentences. Most of the type frequencies showed skewed distributions, and thus log-transformed (base *e*). Following Kuperman and Van Dyke (2011, 2013) and other standard practice, we excluded words with a high likelihood of being skipped (i.e., highly-frequent and very short words).

Table 5 shows the lexical properties of the words contained in the sentences. Regression models targeting online reading indicators (gaze measures) at a word also included parameters for length and frequency of the previous and subsequent words. Differences between Len_*W*_ and Len_*W–1*_ (or Len_*W*+1_) are due to the exclusion of sentence initial and final words in the current word set (see Eye-movement measures), so are differences between Freq_*W*_ and Freq_*W–1*_ (or Freq_*W*+1_).

**TABLE 5:** Lexical properties of the words in the sentence stimuli.

Name	Label	Mean	SD	Min.	25%	50%	75%	Max.	Skew	Kurtosis
Current word length	Len_W_	6.03	2.03	2	4	6	7	14	0.66	0.37
Current word frequency	Freq_W_	10.16	1.85	3.33	9.03	10.35	11.48	13.91	-0.75	1.03
Previous word length	Len_W–1_	4.15	2.12	1	3	3	6	13	0.96	0.68
Previous word frequency	Freq_W–1_	13.50	2.93	4.53	11.15	14.03	16.28	17.04	-0.52	-0.59
Next word length	Len_W+1_	4.19	2.19	1	2	4	5	13	0.99	0.57
Next word frequency	Freq_W+1_	13.09	2.78	3.66	11.00	13.56	15.28	17.04	-0.55	-0.48

Word positions in sentences are excluded here. Word lengths are raw values before mean-centered. Word frequencies are log-transformed type frequencies from the COCA database.

Prior to the analyses, we mean-centered lexical properties. Word length was measured in terms of number of characters. Log-transformed word frequency was standardized. Word frequencies were highly correlated with lengths of respective words: Pearson’s *r* between current word length and current word frequency was −0.731 (*p* < 0.001), −0.775 (*p* < 0.001) between previous word length and previous word frequency, and −0.752 (*p* < 0.001) between next word length and next word frequency. Following Kuperman and Van Dyke (2011), we residualized word frequencies against lengths of respective words. This was done by fitting a regression model for each of the three properties (previous, current, and next words) in which the frequency of the relevant word was predicted by its length. We took the residuals (distances between the observed and fitted values) of these models as the values of word frequency. The residualized frequencies remained strongly correlated with the original frequencies but orthogonal to the lengths of respective words: Pearson’s *r* between residualized and original frequencies was 0.697 (*p* < 0.001) for current word frequencies, 0.643 (*p* < 0.001) for previous word frequencies, and 0.665 (*p* < 0.001) for next word frequencies. The residualization (or orthogonalization) procedure does not change the result for the residualized variable, the overall explanatory power of the model, and any indices of model fit. Some scholars pointed out that such orthogonalization (Wurm and Fisicaro, 2014) could not be a useful remedy for collinearity; note that in our experiment, the significant factors reported by the regression analyses using the orthogonalized or unorthogonalized word frequency and word length values are the same.

### 3.4. Apparatus and procedure

During the test session, participants were instructed to read, one by one, a number of sentences, and to answer yes/no comprehension questions about the contents of the sentences just read (see Supplementary Table 1). Comprehension questions occurred immediately after some sentences on about a sixth of trials to ensure that participants stayed focused on the reading comprehension task throughout the session. The mean response accuracy to the comprehension questions was 0.913 (SD = 0.067).

Each sentence was presented on a single line vertically centered on a monitor, which was positioned approximately 64 centimeters from the participants’ eyes. The sentences were displayed in a monospace font (Bitstream MonoSpace 821) in black with a light background, at a screen resolution 1,280 × 1,024 and a refresh rate 85 Hz. Font size was set such that each character subtended about 17 minutes of visual arc. Participants wore an EyeLink II head-mounted eye tracker (SR Research), the sampling rate of which was set to 250 Hz. Before the test session, the accuracy of the eye tracker was calibrated based on a 9-point full-screen calibration. Over the course of the session, measurement accuracy was monitored, and if needed, the device was re-calibrated (this was rarely necessary). Data were collected binocularly. Our analyses were based primarily on the right eye data. The right eye data of one participant was problematic, and therefore, the left eye data of the participant were used.

In each trial, a fixation point appeared first at the position of the second character of the first word of the sentence (vertically centered on the screen and about 1.5 inches from the left edge). After fixating on this point, participants pressed a button to bring up a sentence and started to read it. Sentences would not show up if participants were not fixating on this point. After reading the whole sentence, participants clicked the button again. This prompted either the next trial or the display of a comprehension question. Participants gave answers to the comprehension questions by pressing the buttons denoting “yes” and “no,” respectively.

### 3.5. Eye-movement measures

We calculated the eye-movement measures using the in-house software (Braze, 2005), which served to tally gaze measures for each word. We removed fixations shorter than 50 ms, as well as blinks and instances of track-loss. We also excluded the sentence initial and final words from analysis, as a common practice (Kliegl et al., 2004). There remained a total of 15,733 eye-movement observations, covering 358 word types in 72 sentences. The volume of the data is comparable to other eye-tracking studies of individual differences. We focused on five informative, widely-used eye-movement measures (Rayner, 1998):

(1) *First fixation duration*, the duration of the initial fixation a reader makes on a region (word) during first-pass reading. It is typically considered to reflect early stage processes during lexical access (Inhoff, 1984).
(2) *First-pass reading time* (a.k.a. *gaze duration*), the summed duration of all fixations a reader makes on a word before fixating any subsequent word, and before gaze leaves the word for the first time, whether advancing to the next word or regressing to an earlier word. It is often considered to reflect sentence structure, parsing decisions (Rayner et al., 1983; Ferreira and Clifton, 1986), or predictability of words in context (Boston et al., 2008). First fixation duration and first-pass reading time are conditional upon a word receiving a first-pass reading. If a word was initially skipped and thus nominally accrued a zero value for these measures, then that data point was omitted from the following analyses, because we do not wish to infer from word-skipping that a word is not processed at all, or that its processing load is zero (Rayner and Pollatsek, 1989).
(3) *Total reading time*, the sum of all fixations falling again into the current word region. It reflects the integrative effect of both early and late stage processes during lexical access.
(4) *Incidence of first-pass regression*, coding for whether the eye-movement at the end of first pass reading moved back to a previous part of the sentence (= 1), or advanced to a subsequent word (= 0).
(5) *Refixation incidence*, being 1 if a word is refixated after the first-pass, or 0 otherwise.

Measures (1) – (3) are continuous, and (4) and (5) are binary (0/1) to capture possible effects on late stage of processing. Measures (4) and (5) are generally treated as indices of processing load associated with integration difficulty (Rayner et al., 1983, 1989).

Table 6 summarizes the gaze measures, which reflect different, but perhaps overlapping stages of word recognition, text comprehension, and integration during online sentence reading. First fixation duration and first-pass reading time reflect the early stages of print processing involving first encounter of a word by the reader following the default reading direction (left to right in English). By contrast, incidence of first-pass regression and refixation incidence reflect the later stages of print processing involving integration of word information with syntactic and/or discourse context or resolution of ambiguity whenever necessary (Vasishth et al., 2013). Total reading time is a cumulative index of “early” and “late” stages of processing. Individual differences in several components of skilled reading (e.g., decoding, oral reading fluency, vocabulary knowledge, working memory) may have different effects as gauged by these eye-movement measures (Kuperman and Van Dyke, 2011; Nelson Taylor and Perfetti, 2016).

**TABLE 6:** Summary of the eye-movement measures.

Name	Mean	SD	Min.	25%	50%	75%	Max.	Skew	Kurtosis
First fixation duration	236.98	96.79	52	176	216	272	996	1.92	6.54
First-pass reading time	293.54	145.47	52	192	252	360	1000	1.50	2.73
Total reading time	378.07	237.39	52	212	312	464	3080	2.28	9.39
Incidence of first-pass regression	0.16	0.37	0				1	1.80	1.25
Refixation incidence	0.25	0.43	0				1	1.14	-0.70

The first three measures are continuous, and the other two are binary (0/1).

In our dataset, 11,965 out of the total 15,733 eye-movement observations (76.050%) were first-pass eye-movements, and only 3,768 had distinct first fixation durations and first-pass reading times. This indicates that during first-pass reading, most words were fixated exactly once (many words in our simple stimuli sentences were short; see Table 5, over half of the words are shorter than 6 characters). Therefore, it is expected that if any factors can exert significant effects during first-pass reading, they might be captured mainly by first-pass reading time, not by first fixation duration. In addition, our stimuli sentences were simple in structure, which might not trigger many regressive eye-movements or second-pass reading in our participants. Therefore, incidence of first-pass regression and refixation incidence might not capture many significant effects, unlike previous studies involving more complex sentence stimuli (Kuperman and Van Dyke, 2011; Kuperman et al., 2018)).

### 3.6. Analytic approach

We conducted two types of statistical analysis.

First, we used linear and logistic mixed-effects regression models (Baayen, 2008; Quené and van den Bergh, 2008) with crossed random effects to analyze respectively the continuous and categorical eye-movement measures and identify interactions between lexical properties and reading related skills. Mixed-effects models allow for simultaneous consideration of multiple covariates, while keeping the between-participants and between-items variance under statistical control (Pinheiro and Bates, 2000; Baayen et al., 2008). Unlike the random forest models used in Kuperman et al. (2018), mixed-effects models can simultaneously address multiple factors having different scales of effect sizes and directly report significance of main effects and/or interactions.

We fit five mixed-effects regression models (Quené and van den Bergh, 2008) targeting the five eye-movement measures, respectively. To reflect the collinearity of a model, we reported the condition number kappa of the model and the maximum variance inflation factor (VIF) of all predictors in the model. A condition number kappa smaller than 10 and a VIF smaller than 5 typically indicate a low degree of collinearity (Kutner et al., 2004).

Each of the five models included 23 fixed effects, consisting of seven lexical properties, four composite and single skill measures, and 12 interactions between each of the skill measures and each of the lexical properties, namely word position in a sentence, word frequency and word length. This approach provides an integrative picture of the effects of multiple skill measures on eye-movement patterns. We controlled the family-wise Type I error probability by setting the critical *p* value for identifying significance as 0.05/23 ≈0.00217. Given this extremely strict setting of critical *p* value, we focused on both the significant (*p* < 0.00217) and marginally significant (*p* is close to 0.00217) factors.

Each model included the same random effect structure, consisting of two intercepts respectively for subject and for word nested under sentence, and one slope of word frequency for subject. In principle, the slope of word length for subject should also be added in each model. However, as shown above, word length was negatively correlated with word frequency, and post-hoc analyses revealed that the separate contributions of word length to the variation in the dependent variables was <1%. Therefore, we excluded this slope in the regression models. In addition, maximal random effect structures involving other types of slopes are theoretically desirable (Barr et al., 2013) and have been applied in recent individual difference studies (e.g., Protopapas and Kapnoula, 2016). However, we did not pursue such complicated models in consideration of practical constraints on model convergence (Bates et al., 2015a).

All the mixed-effects models were implemented using the R packages *lme4* (Bates et al., 2015b) and *lmerTest* (Kuznetsova et al., 2017).

Second, after identifying significant interactions, we continued examining the dynamics of lexical properties and eye-movement measures in individuals having different levels of target skills. Very few existing studies have investigated such dynamics. Our approach proceeded as follows. Given a two-way interaction between a lexical property and a skill measure, we first divided the participants into a high and a low group based on the medium value of the skill measure to ensure the same number of participants in each group. Then, we plotted the eye-movement measure in each group against the lexical property. A cross-group comparation of the correlations between lexical properties and eye-movement measures could reveal the effects of individual skill on online reading behavior. Instead of binary groups, quartile or quintile groups were used in some studies (e.g., Protopapas and Kapnoula, 2016), given enough participants in each group for statistical analysis. To identify correlation, we first fit a nonlinear polynomial regression (*loess*) between the lexical property and the eye-movement measure as the baseline, and then, used widely-adopted regression models in psychological and educational research to quantify the pattern of the correlation. For simplicity, the current study only compared simple linear regression (or logistic regression) and segmented linear regression. For each model, lexical property was treated as an independent variable, and eye-movement measure a dependent one.

Models were compared based on Akaike information criterion (AIC) and mean squared error (MSE). AIC deals with the trade-off between the simplicity and goodness of fit of a model (Akaike, 1974), but AIC alone is less informative when multiple models have similarly high or low AICs (Burnham and Anderson, 2002). In this situation, MSE is referred to, which compromises variance and bias to minimize both (see Equation 1, where *obs*_*i*_ is the observed essay score, *pre*_*i*_ is the predicted score from a model, and *n* is the number of data points). The *best* model that appropriately reflects the correlation between lexical property and eye-movement measure is the one having smaller AIC and MSE.


(1) $$M\,S\,E=\frac{1}{n}\,\sum_{i=1}^{n}{\left(o\,b\,s_{i}-p\,r\,e_{i}\right)}^{2}$$

A recent study examining the correlation between typing speed and writing essay score has used a similar method to identify the dynamics of such correlation (Gong et al., 2022). In that study, additional models like logistic regression and ordinal categorical regression were used for model fitting, but the segmented linear regression remained the best fitting model.

In our study, the segmented regression was implemented using the R package *segmented* (Muggeo, 2008).

## 4. Results

The analyses were carried out in R 3.2.4 (R Core Team, 2013). The raw data, R codes, and the results can be found at: https://github.com/gtojty/IndDiff_EM.

All the regression models showed a low degree of collinearity; the kappas of these models were all below 10 and the VIFs of the independent factors in these models were all below 5. The significant main effects of lexical properties reported in these models are shown in Supplementary Table 2 and discussed in Supplementary material. No skill measures showed significant main effects on any eye-movement measures (their *p* values were all above. 00217), due primarily to the wide spans of the skill measures in our study.

**FIGURE 1:**
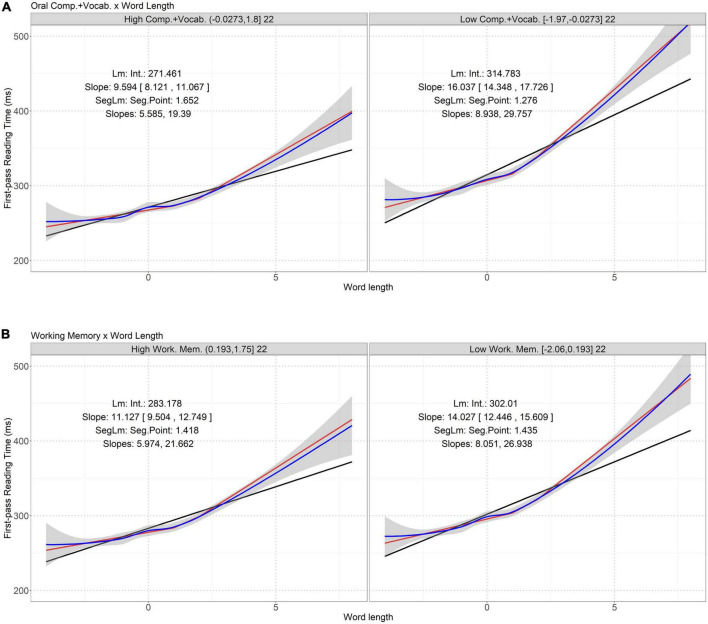
Interactions between word length and oral comprehension plus vocabulary **(A)** and verbal working memory **(B)** on first-pass reading time. Word length is mean-centered. The two panels in each figure represent the high and low skill groups. The titles of the panels show the level ranges (within round or square brackets) of the skill measure in the two groups and the numbers of participants in these groups. In each panel, the blue line is the loess fitting curve and the shaded area is standard error. The black line is the linear regression fitting curve (“Lm”). “Int.” shows the interception (β_0_), and “Slope” the slope (β_1_). Numbers in square brackets are 95% confidence interval of the slope. The red line is the segmented linear regression fitting curve (“SegLm”). “Seg.Point” shows the pivot point at word length, below and above which the slopes of the curve are distinct (see “Slopes”). See Supplementary Table 3A for AIC and MSE of these models. The segmented linear models have the smallest AIC and MSE closest to that of the loess regressions.

**FIGURE 2:**
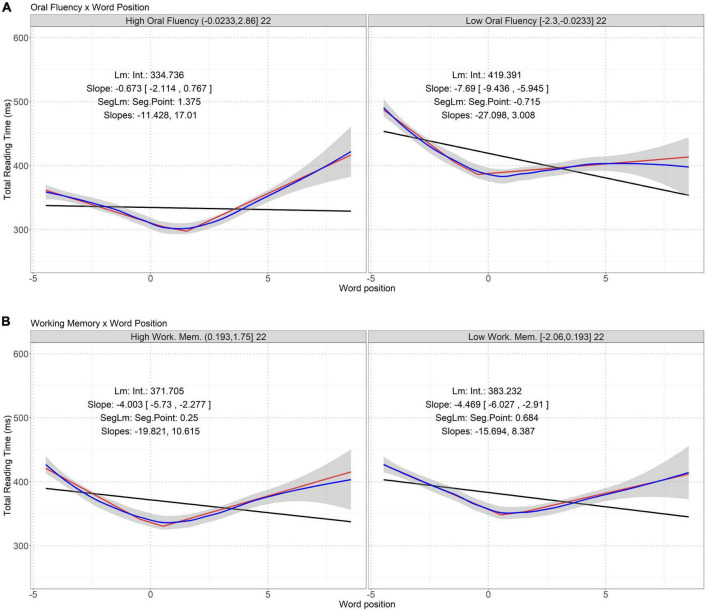
Interactions between word position and oral fluency **(A)** and verbal working memory **(B)** on total reading time. Word position is mean-centered. The two panels in each figure represent the high and low skill groups. See Supplementary Table 3B for AIC and MSE of different models, which shows the segmented linear models have the smallest AIC and MSE closest to that of the loess regressions.

**FIGURE 3:**
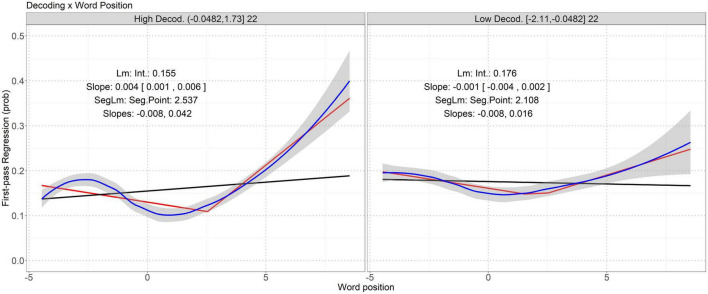
Interaction between word position and decoding on incidence of first-pass regression. Word position is mean-centered. The two panels represent the high and low decoding groups. See Supplementary Table 3C for AIC and MSE of different models, which shows the segmented linear models have the smallest AIC and MSE closest to that of the loess regressions. “Lm” here is logistic regression. Note that in the left panel, it seems that the loess regression fitting curve also has a pivot point near the lower bound of word position. Since it is much closer to the boundary, there are insufficient data points for the segmented linear model to identify it as a pivot point.

Our study focuses on the interactions whose *p* values are smaller than (significant) or close to (marginally significant) the threshold. 00217. For the sake of completeness, Tables 7–10 list all the interactions between lexical properties and skill measures having *p* values below 0.05/5 = 0.01. Effect size (Cohen’s *d*) of each interaction was measured using the lme.dscore function in the R package *EMAtools*.^2^ Significant (and marginally significant) interactions are visualized in Figures 1–3. For each interaction, the correlation between the involved lexical property and eye-movement measure in the participants having high and low levels of the involved skill measure can be best described as a segmented linear relation. Below, we discuss these interactions identified in the regression models.

**TABLE 7:** Interaction on first fixation duration.

Factor	Est.	SE	*t*	*p*	*d*
Decoding × word frequency	3.995	1.480	2.699	0.009	0.744

Its *p* value is below 0.01 but over 0.00217.

**TABLE 8:** Interactions on first-pass reading time.

Factor	Est.	SE	*t*	*p*	*d*
**Oral comprehension plus vocabulary × word length**	-**2**.**513**	**0**.**846**	-**2**.**970**	**0**.**002**	-**0**.**045**
**Verbal working memory × word length**	**2**.**890**	**0**.**831**	**3**.**480**	**0**.**001**	**0**.**058**

All listed interactions have *p* values below 0.01. Interactions having *p* values below or close to 0.00217 are bolded.

**TABLE 9:** Interactions on total reading time.

Factor	Est.	SE	*t*	*p*	*d*
**Oral reading fluency × word position**	**2**.**013**	**0**.**661**	**3**.**040**	**0**.**002**	**0**.**051**
**Verbal working memory × word position**	-**2**.**196**	**0**.**732**	-**3**.**000**	**0**.**002**	-**0**.**050**
Verbal working memory × word length	3.885	1.353	2.870	0.004	0.048

All these interactions have *p* values below 0.01. Interactions having *p* values below or close to 0.00217 are bolded.

**TABLE 10:** Interaction on incidence of first-pass regression.

Factor	Est.	SE	*z*	*p*	*d*
**Decoding × word position**	**0.031**	**0.009**	**3.276**	**0.001**	**0.055**

Its *p* value is below 0.00217. Statistically significant factors are shown in bold.

### 4.1. First fixation duration

Table 7 lists one interaction between word frequency and decoding skill in determining first fixation duration. Its *p* value is over .00217, so it is not marked as a significant interaction.

### 4.2. First-pass reading time

Table 8 shows two interactions on first-pass reading time whose *p* values are below .01. Given their *p* values are smaller than (or close to) .00217, they are marked significant (or marginally significant). Figure 1 illustrates these interactions by showing that the correlation between word length and first-pass reading time is contingent on oral comprehension plus vocabulary and verbal working memory.

Figure 1 shows that the sensitivity of first-pass reading time to word length is better described as a segmented linear relation than a simple linear relation: the segmented linear curves well match the baseline loess curve and have smaller AIC and MSE than the linear curve (see Supplementary Table 2). In each panel, the segmented linear curve shows a pivot value of word length, below which the slop of the fitting curve remains small, whereas above which the slope increases, indicating that the participants showed longer first-pass reading time when reading longer words. Between the two panels in each figure, the sensitivity of first-pass reading time to word length exhibits different tendencies.

In Figure 1A, compared to the poor readers having low levels of oral comprehension plus vocabulary (the right panel), for words of the same length, the good readers having high levels of that skill (the left panel) had shorter first-pass reading time. Also, the good readers showed smaller slopes in the segmented linear curve than the poor readers (i.e., 5.585 vs. 8.938 and 19.39 vs. 29.757), indicating that the good readers were less sensitive to word length. Finally, the pivot points of word length were similar in the poor (1.652) and good (1.276) readers.

In Figure 1B, similarly, compared to the good readers having high levels of verbal working memory, the poor readers having low levels of that skill spent relatively more time in reading long words, and for both long and short words, their first-pass reading times remained more sensitive to word length (shown by the slopes of the segmented linear curves, 26.938 vs. 21.662 and 8.051 vs. 5.974). Nonetheless, the pivot points of word length in the poor and good readers were similar (1.435 vs. 1.418).

### 4.3. Total reading time

Table 9 shows three interactions on total reading time whose *p* values are below .01, two of which are marked as marginally significant and visualized in Figure 2.

Figure 2 illustrates a segmented linear relation between total reading time and word position in a sentence. Total reading time drops when the participants read the first few words in a sentence, and then, increases when they read the latter words in a sentence. The negative and positive slopes of the segmented linear fitting curves clearly reflect this bifurcating tendency.

In Figure 2A, compared to the good readers having high levels of oral reading fluency, the total reading time of the poor readers having low levels of that skill is generally longer, and it is more sensitive to the beginning words in a sentence, as shown by the more negative slopes (−27.098 vs. −11.428) below the pivot points of word position. However, the smaller positive slopes (3.008 vs. 17.01) above the pivot points suggest that the total reading time of the poor readers is less sensitive to the latter words in a sentence. In addition, the pivot points of word position increases from −0.715 in the poor readers to 1.375 in the good readers.

In Figure 2B, compared to the good readers having high levels of verbal working memory, the total reading time of the poor readers having low levels of that skill is less sensitive to word position in a sentence, as shown by the smaller absolute slopes both below (−15.694 vs. −19.821) and above (8.387 vs. 10.615) the pivot points of word position. In addition, the pivot points in the two panels drop from 0.684 in the poor readers to 0.250 in the good readers.

A comparison of Figures 1, 2 reveals that verbal working memory casts its influence on first-pass reading time via interaction with word length and total reading time via interaction with word position. To be specific, compared to the poor readers having low levels of verbal working memory, the first-pass reading time of the good readers is less sensitive to word length, but their total reading time is more sensitive to word position.

### 4.4. Incidence of first-pass regression

Table 10 shows that the interaction between decoding and word position had a *p* value below .00217. Figure 3 visualizes this significant interaction.

Figure 3 shows a segmented linear relation between first-pass regression and word position in a sentence. The probability of regression during the first-pass reading starts to increase when the participants read the latter words in a sentence. Compared to the poor readers having low levels of decoding, the probability of regression during the first-pass reading of the good readers increases a lot on the latter words in a sentence, as shown by bigger slopes (.042 vs. .016) above the pivot points of word position. The pivot points of word position are similar in the poor (2.108) and good (2.537) readers.

### 4.5. Refixation incidence

No interactions on refixation incidence have *p* values below .01.

## 5. Discussion

### 5.1. Effects of interactions between language and literacy skills and lexical properties on online reading behavior

Previous studies have reported significant main effects of some of the language and literacy skills discussed in this paper, or bigger effect sizes of these skills than those of lexical properties (e.g., Kuperman and Van Dyke, 2011). However, in our analyses, main effects of skill measures never reach statistical significance, though those of lexical properties often do. The effect sizes of the skill measures are also smaller than those of lexical properties. This is because that our study focused on individuals with a much wider range of language and literacy skills; only those having the highest scores of the skill measures were comparable to university students (cf. Braze et al., 2007). Such wide range of individual differences in the skill measures could result in insignificance and low effect sizes of the measures on online reading behavior. These findings can enrich existing evidence and trigger revisits on the theoretical discussions of individual differences and their roles in reading process and outcome (comprehension) (Bennink and Spoelstra, 1979; Bleckley et al., 2003).

Although lacking direct influence on online reading behavior, some of the language and literacy related skills could significantly influence online reading behavior via interactions with lexical properties. Our study showed that oral comprehension, vocabulary, verbal working memory, oral reading fluency, and decoding could predict online reading patterns via interactions with word length or position in a sentence. We also compared the effects of the interactions involving these skills on online reading patterns between the good and poor readers with respect to these skills.

To be specific, oral comprehension and vocabulary interact with word length to predict first-pass reading time (see Figure 1A); readers with good oral comprehension skill and vocabulary knowledge could efficiently process words with various lengths, thus being less troubled by long words during first-pass reading. First-pass reading time arguably reflects the duration of lexical processing, including recognition of orthographic or phonological features of a word and retrieval of semantic information from memory once attention is allocated to the word (Inhoff, 1984). This finding contributes to recent discussions on whether vocabulary knowledge could influence reading comprehension over and above the effect of language comprehension including listening comprehension (Braze et al., 2007, 2016; Tunmer and Chapman, 2012; Protopapas et al., 2013). At the early stage of print processing vocabulary knowledge already helps good readers efficiently reduce first-pass reading time on words of various lengths.

Verbal working memory presumably affects the rate at which word information is assimilated during first-pass reading, especially on long words. As shown in Figure 1B, the first-pass reading time of the good readers with high levels of verbal working memory are less sensitive to word length than the poor readers. In addition, verbal working memory helps predict total reading time via interaction with word position (see Figure 2B). Total reading time reflects the integration of early and late processing during lexical access. Word position in a sentence is a context-dependent property. A general increase in total reading time on words toward the end of a sentence reflects so-called wrap-up effects (Rayner et al., 2000; Warren et al., 2009). In our study, such effects became more explicit in readers having high levels of verbal working memory; efficient verbal working memory reduces the processing time for the first few words of a sentence but induces more wrap-up effects towards the end of a sentence.

Oral reading fluency interacts with word position to predict total reading time (see Figure 2A); a high level of this skill is associated with a less sensitivity to the first few words in a sentence, but more sensitivity to latter words in a sentence, in line with the wrap-up effects. In addition, less fluent readers generally have more difficulty in processing individual words and integrating word semantics with context, and hence spend more time reading a few words of a sentence; by contrast, more fluent readers spend less time reading words in a sentence, especially those near the beginning or in the middle of a sentence. These findings are in line with and complement the existing theories on oral and/or silent reading fluency (Fuchs et al., 2001; Tilstra et al., 2009; Kim et al., 2011; Silverman et al., 2012; Ashby et al., 2013). Furthermore, as shown in Figure 2, there is no monotonic change of the correlation between word position and total reading time. This indicates that the effects of oral reading fluency and verbal working memory on regulating online reading patterns are complex, possibly also subject to other factors.

Decoding skill interacts with word position to predict probability of first-pass regression (see Figure 3); good decoders tended to have more regressive reading when reading words towards the end of a sentence, reflecting their sentence decoding processes. Early studies have reported the effects of decoding on early (first-pass reading time) and overall (total reading time) reading and re-reading probability (Kuperman and Van Dyke, 2011; Nash and Heath, 2011; Kuperman et al., 2018). In our study, the effect of decoding on re-reading probability was fulfilled via an interaction with word position. All these are in line with the claims that decoding skill is among the key factors in lexical access (Barth et al., 2009; Hulme and Snowling, 2012) and provide evidence for VET (Perfetti, 1985; Shankweiler and Crain, 1986) and LQH (Perfetti and Hart, 2002; Perfetti, 2007) by showing how decoding influences reading processes.

### 5.2. Segmented linear dynamics of the correlation between lexical properties and eye-movement measures

In addition to confirming that language and literacy skills can influence online reading behavior indirectly via interactions with lexical properties, our study further investigated the dynamics of the correlation between lexical properties and eye-movement measures regulated by particular individual skills. Our quantitative analyses revealed that such dynamics cannot be simply described as a linear relation; instead, many of the correlations follow a segmented linear relation, with at least two distinct slopes throughout the values of the relevant lexical properties. Some of the dynamics are monotonic (see Figure 1), with positive and increasing slopes around long words, whereas others are not (see Figures 2, 3), with a transition from a negative to a positive slope. The observed segmented linear relations suggest a complex effect of key language and literacy skills on regulating reading patterns via interactions with word length or position. Between the good and poor readers based on some skills, the durations of reading time are different, so are the sensitivity of reading time or regression probability to word length or position. In addition, the pivot values of word length or position in the segmented linear correlations indicate a transition of the degree of correlation. Note that in many cases, the pivot points are not close to the mean value 0, so arbitrary binary segmentation based on word length or position (Kuperman et al., 2018) cannot clearly reveal such dynamics. This dynamics echoes the effects of interactions between lexical properties and skill measures on online reading behavior: due to individual skills, the unimodal associations between eye-movement patterns and lexical properties are broken, the degrees of associations become different when the values of lexical properties are below or above the pivot points, and the high and low levels of the skills further influence the pivot lexical property values and the degrees of associations below and above the pivot values.

The observed dynamics in all these aspects can lead to more comprehensive theories on the dynamic relations between individual skills, text properties, and reading process. For example, some theories of reading (Perfetti and Hart, 2002) and empirical studies (Johnston and Kirby, 2006; Savage, 2006) have challenged the linear assumption between decoding and reading outcomes like reading comprehension. For example, Johnston and Kirby (2006) showed that naming speed, a measure of decoding skill, had its primary effect on less able readers. A recent study of reading assessment has shown distinct relations between decoding skill and comprehension scores between good and poor decoders in Grades 5 to 10 (Wang et al., 2019). Some eye-movement studies have revealed close relations between components of decoding (e.g., phonemic awareness) and other skills (e.g., reading fluency) (Barth et al., 2009; Ashby et al., 2013). Our study enriched the findings in this line of research by visualizing the segmented linear relations between lexical properties and online reading behavior, which are manipulated by individual differences in individual language and literacy skills. This study can also inspire more empirical studies to further investigate what factors help shape the slopes and pivot values in the segmented linear models.

## 6. Conclusion

This study investigated the eye-movement data of simple sentence reading from 44 young adults in high schools, adult education centers, community colleges, or neighborhood communities. A total of six domains of individual differences, plus age, were tested to assess their effects via themselves and interactions with lexical properties on online reading behavior. Three of these domains tap into components of reading ability: reading comprehension, decoding skill, and oral reading fluency. The other three tap into domains not reading specific: listening comprehension, vocabulary, and verbal working memory. By evaluating the effect of each domain while controlling for the others, we identified a series of interactions between properties of text (length and position) and skills of readers (oral comprehension, vocabulary, verbal working memory, oral reading fluency, and decoding), which manipulated both the early and late stages of online reading process as gauged by eye-movement measures (first-pass reading time, total reading time, and first-pass regression). We also visualize segmented linear dynamics of the effects of these interactions on online reading patterns. All these findings speak to the necessity of incorporating interactions between lexical properties and reading-related skills to enrich empirical evidence, extend and refine theories about reading outcomes and processes, and trigger new theories or hypotheses on how language and literacy skills interact with lexical properties to influence reading process.

## Data availability statement

The datasets presented in this study can be found in online repositories. The names of the repository/repositories and accession number(s) can be found below: https://github.com/gtojty/IndDiff_EM.

## Ethics statement

The studies involving human participants were reviewed and approved by Haskins Laboratories. Informed consent was obtained from the participants of at least 18 years old; for those under 18, the participants provided assent and their parents or guardians signed written permissions.

## Author contributions

TG designed and implemented the experiment. Both author collected and analyzed the data and wrote and edited the manuscript.

## Footnotes

1
http://corpus.byu.edu/coca/

2
https://cran.r-project.org/web/packages/EMAtools/index.html
