# Supplementary material for: Segmented relations between online reading behaviors, text properties, and reader–text interactions: An eye-movement experiment
Source: Front Psychol. 2023 Jan 11;13:1006662. doi: 10.3389/fpsyg.2022.1006662 (PMC9874682; doi:10.3389/fpsyg.2022.1006662)
Supplement: Supplementary file 1 [file Data_Sheet_1.docx]

**Segmented Relations between Online Reading Behaviors, Text Properties, and Reader-Text Interactions: An Eye-Movement Experiment**

**Supporting Material**

**Table S1.** The 72 sentence stimuli and the comprehension questions about some of these sentences used in our study. Sentences marked by “*” contain embedded clauses.

| **Index** | **Sentence** | **Comprehension question** | **Ans.** |
| --- | --- | --- | --- |
| 1 | It's a custom in the United states to eat turkey for dinner on Thanksgiving. |  |  |
| 2* | If the drought continues for much longer all of the farmer's crops will be ruined. |  |  |
| 3 | Most of the students will be going to the class picnic next month. | Is anyone going to the picnic? | Yes |
| 4* | Jennifer wanted cake but by the time she arrived there was only pie left. |  |  |
| 5 | There have been very few qualified applicants for the new position. |  |  |
| 6* | Our congressman said that he was aided by the efforts of many volunteers. | Did he get any help? | Yes |
| 7 | Several hundred college students were called at random by the pollster. |  |  |
| 8* | The waiter had told the customer that the pies were fresh. |  |  |
| 9 | The new exhibit at the museum features more than fifty original paintings. |  |  |
| 10 | A series of sports camps will be available this summer for inner-city children. |  |  |
| 11 | Jim saw two black puppies chasing a cat in the yard this morning. |  |  |
| 12 | One of the lawyer's best clients always paid his bills early. |  |  |
| 13 | There was an ashtray on the coffee table but nobody used it. |  |  |
| 14* | Tom had an accident because he was driving way too fast on the freeway. | Is Tom a good driver? | No |
| 15* | The price of heating oil is very high because it is so cold this winter. |  |  |
| 16* | Nobody knew why the speaker always concluded each lecture with a bad joke. |  |  |
| 17 | Harry sets a little money aside every month to pay for his daughter's education. |  |  |
| 18 | The policeman was giving directions to a woman at the bus stop. |  |  |
| 19 | They saw few giant ferns growing in the remnants of the rain forest. | Is the rain forest thriving? | Yes |
| 20* | The parents spoke to their daughters to find out what really took place at the party. |  |  |
| 21 | The weather forecast for tomorrow predicts thunderstorms hail and high winds. |  |  |
| 22 | We had decided to call the office for information about financial aid programs. |  |  |
| 23 | The programmer was designing a computer game for the company to sell. |  |  |
| 24* | Rats quickly learn not to eat food that has caused them to be ill. |  |  |
| 25 | Jill wants all violent criminals to get the harshest possible penalties. | Should the penalties be stiff? | Yes |
| 26* | Young artists often go hungry because they have trouble earning a proper wage. |  |  |
| 27* | Sally was relieved when she found out the answer to the extra-credit problem. |  |  |
| 28 | Bill argued with Fred for an hour before deciding to leave the room to cool off. |  |  |
| 29* | The student missed the point completely because her teacher didn't get it across well. |  |  |
| 30 | One reporter was caught trying to sneak a hidden camera into the courtroom. |  |  |
| 31 | George needs to call his insurance agent about the break-in last night. |  |  |
| 32 | Debbie caught two unfortunate rats and five mice in her house yesterday. | Was the house infested? | Yes |
| 33* | Every student is required to write a report that is at least ten pages long. |  |  |
| 34* | I wonder if tom has heard the latest gossip about the new neighbors. |  |  |
| 35 | The program director used to write letters but now uses the phone. |  |  |
| 36* | Jody picked several ripe oranges for the fruit salad she was making. | Did she use oranges? | Yes |
| 37 | Clean drinking water can be hard to find in tropical places. |  |  |
| 38 | Toni found two antique bottles on a shelf in the back of the storage shed. | Was Toni in the shed? | Yes |
| 39* | someone forgot to turn off the lights when they left last night |  |  |
| 40 | The van stolen from the parking lot belongs to the hardware store next door. |  |  |
| 41* | Most athletes will follow a strict diet when they are in training. |  |  |
| 42* | The sailor saw three of his crewmates sneak off the ship after curfew. |  |  |
| 43 | Albert Einstein expressed the principles of relativity in very simple terms. |  |  |
| 44 | A little yellow dog was slowly trotting along the dusty country road. | Was it raining? | Yes |
| 45* | Li really hopes that Gerry will come to the party tonight. |  |  |
| 46 | The bellboy dropped the suitcase twice on the way up to the room. |  |  |
| 47 | The two countries broke off diplomatic relations over the border incident. |  |  |
| 48 | Travelers will sometimes have to pay a surcharge on excess baggage. |  |  |
| 49* | Goldfish are the only pets we are allowed to keep in our apartment. |  |  |
| 50 | Our neighbors appeared relaxed after their two-week vacation in Florida. |  |  |
| 51 | The article rebuts some untrue claims about the senator's past behavior. | Does the article prove the claims? | Yes |
| 52* | All the people at the resort were glad to see the sun finally come out. |  |  |
| 53 | We were very excited to see the huge python at the zoo. |  |  |
| 54* | If you move the chairs away from the table you should put them back later. |  |  |
| 55 | Those three authors each decided to send a manuscript to the new editor. |  |  |
| 56 | The newly drilled well is spouting an enormous black geyser of oil. |  |  |
| 57* | The farmer had warned the stranger that a tornado was on the way. |  |  |
| 58 | The breakdown on the subway is going to make me late for work again. |  |  |
| 59 | The applications for admission sometimes take several weeks to be processed. |  |  |
| 60* | Every time it rains the duck pond will overflow onto the grass. |  |  |
| 61 | The press coverage of the incident did not please Jim at all. |  |  |
| 62 | They always put a carved wooden angel on top of their Christmas tree. | Is the angel made of glass? | No |
| 63 | It is very easy for a clever criminal to pass off bad checks. |  |  |
| 64 | All valid bids for this project must be postmarked before the end of November. |  |  |
| 65* | Cats sometimes act as if they are angry with their owners. |  |  |
| 66 | Sandra is mixing the leafy vegetables with tomatoes to make a dinner salad. | Are there peppers in the salad? | No |
| 67 | Several students arrived early enough to have some coffee before class began. |  |  |
| 68 | Our Bible study group will meet next Wednesday to discuss the trials of job. |  |  |
| 69 | The young woodsman had remembered to take a heavy wool jacket on his hike. |  |  |
| 70 | The well water in this area is too badly polluted to be used. |  |  |
| 71 | Doug will fight to remain chairman of the company despite the stockholders' opposition. |  |  |
| 72* | Henry is afraid that he will be deported because his visa has expired. |  |  |

**Significant main effects reported by the mixed effects regression models**

Tables S2 summarizes the main effects having *p* values below .05/5 = .01 reported by the models respectively for the five gaze measures. Significant main effects (shown in bold) have *p* values below the critical *p* value of .00217.

These tables reveal the contributions of lexical properties to eye-movement patterns. First, word position in a sentence influenced first fixation duration, first-pass reading time, and probability of refixation. First fixation duration and first-pass reading time increased toward the end of a sentence, which reflected the start-up (gradual increase in reading times as the eye departs from the beginning of a sentence and moves downstream) and wrap-up (increase in reading times towards the end of a sentence) inflations of reading times (Kuperman, Dambacher, Nuthmann, & Kliegl, 2010; Warren, White, & Reichle, 2009), though the sentence initial and sentence final words were excluded in our study. However, as shown in Figure 2, change in total reading time along with word position is not constant; instead, it follows two distinct slopes as indicated by the segmented linear fitting curve. This reflects that in addition to the lexical property of word position, other factors from individual skill measures could also influence total reading time, e.g., oral reading fluency and verbal working memory. In addition, probability of refixation dropped toward the end of a sentence.

Second, words with higher frequencies (Freq_W_) came with reduced durations of first fixation duration, first-pass reading time, and total reading time. Increase in previous word frequency (Freq_W-1_) reduced total reading time and probability of refixation. Increase in next word frequency (Freq_W+1_) reduced durations of first-pass reading time, total reading time, and probability of refixation of current words.

Third, longer words with more characters (Len_W_) came with longer durations in first-pass and total reading times. However, as shown in Figure 1, change in first-pass reading time follows a segmented linear fashion by showing two slopes. This also suggests that in addition to the lexical property of word length, some skill measures (e.g., oral comprehension plus vocabulary and verbal working memory) also influence the correlation between word length and first-pass reading time. In addition, longer words also increased probability of refixation. Longer previous words (Len_W-1_) reduced total reading time, which reveals the spillover effect from preceding words to current words (Henderson & Ferreira, 1990; Kennison & Clifton, 1995; Kuperman et al., 2010; Rayner & Duffy, 1986). Longer previous words also decreased probability of first-pass regression and probability of refixation.

Many of these results replicate previous findings about the main effects casted by lexical properties on eye-movement measures (Joseph, Nation, & Liversedge, 2013; Kliegl, Nuthmann, & Engbert, 2006; Kuperman & Van Dyke, 2011; Pynte & Kennedy, 2006; Rayner, 1998; Rayner, Cook, Juhasz, & Frazier, 2006; Valle, Binder, Walsh, Nemier, & Bangs, 2013). However, the additional dynamics analyses suggest that simple linear mixed-effects models alone may not explicitly reveal the dynamic relations among lexical properties, individual skill measures, and eye-movement patterns.

**Table S2.** Main effects having *p* values below 0.01 in five regression models of eye-movement measures ((a)-(e)). Effects having *p* values below .00217 are bolded.

(a) First fixation duration

| **Factor** | **Est.** | **SE** | ***t*** | ***p*** | ***d*** |
| --- | --- | --- | --- | --- | --- |
| **Word position** | **2.502** | **.3565** | **7.02** | **< .00001** | **.7222** |
| **Freq_W_** | **-8.384** | **1.505** | **-5.57** | **< .00001** | **-.9328** |
| Len_W_ | -2.599 | .6642 | -3.91 | < .0005 | -.4011 |

(b) First-pass reading time

| **Factor** | **Est.** | **SE** | ***t*** | ***p*** | ***d*** |
| --- | --- | --- | --- | --- | --- |
| **Word position** | **1.9304** | **.6050** | **3.19** | **.00154** | **.3289** |
| **Freq_W_** | **-17.6550** | **2.8955** | **-6.10** | **< .00001** | **-1.0818** |
| **Len_W_** | **8.9534** | **1.1332** | **7.90** | **< .00001** | **.8026** |
| **Freq_W+1_** | **-11.3091** | **2.9932** | **-3.79** | **.00018** | **-.3843** |

(c) Total reading time

| **Factor** | **Est.** | **SE** | ***t*** | ***p*** | ***d*** |
| --- | --- | --- | --- | --- | --- |
| Word position | -2.8666 | 1.0538 | -2.72 | .00683 | -.28299 |
| **Freq_W_** | **-29.8104** | **4.8695** | **-6.12** | **< .00001** | **-.98123** |
| **Len_W_** | **12.9602** | **1.9967** | **6.49** | **< .00001** | **.65908** |
| **Freq_W-1_** | **-18.5729** | **5.3624** | **-3.46** | **.00059** | **-.35444** |
| **Len_W-1_** | **-9.8304** | **2.6116** | **-3.76** | **.00019** | **-.38154** |
| **Freq_W+1_** | **-21.5060** | **5.2583** | **-4.09** | **< .0001** | **-.41772** |

(d) First-pass regression

| **Factor** | **Est.** | **SE** | ***z*** | ***p*** | ***d*** |
| --- | --- | --- | --- | --- | --- |
| Freq_W-1_ | -.1882 | .0669 | -2.812 | .00493 | -.26119 |
| **Len_W-1_** | **-.1734** | **.0328** | **-5.295** | **< .00001** | **-.50547** |

(e) Refixation incidence

| **Factor** | **Est.** | **SE** | ***z*** | ***p*** | ***d*** |
| --- | --- | --- | --- | --- | --- |
| **Word position** | **-.0631** | **.0092** | **-6.861** | **< .00001** | **-.72177** |
| **Len_W_** | **.0632** | **.0175** | **3.612** | **.00030** | **.34327** |
| **Freq_W-1_** | **-.1455** | **.0472** | **-3.082** | **.00206** | **-.32537** |
| **Len_W-1_** | **-.1064** | **.0232** | **-4.580** | **< .00001** | **-.47421** |
| **Freq_W+1_** | **-.1610** | **.0458** | **-3.515** | **.00044** | **-.36582** |

**Table S3.** AIC and MSE between different models for selecting the best fitting model to the correlations between lexical properties and eye-movement measures. “loess” refers to the polynomial regression, “lm” refers to linear regression (or logistic regression for binary eye-movement measures), and “seglm” refers to segmented linear regression. In each row of a table, the smaller AIC between “lm” and “seglm” and smallest MSE are bolded. In all cases, the segmented linear regression model is the best fit model that has smaller AIC and MSE closest to that of the polynomial regression model (in many cases, the segmented linear regression model also has the smallest MSE).

1. First-pass reading time

| **skill** | **Group** | **AIC_loess** | **AIC_lm** | **AIC_seglm** | **MSE_loess** | **MSE_lm** | **MSE_seglm** |
| --- | --- | --- | --- | --- | --- | --- | --- |
| Oral comprehension plus vocabulary | high | NA | 96308 | **96287** | 24.597 | 75.942 | **21.744** |
|  | low | NA | 103960 | **103917** | 30.265 | 157.221 | **23.678** |
| Verbal working memory | high | NA | 89204 | **89180** | 39.431 | 109.390 | **35.452** |
|  | low | NA | 111537 | **111500** | 42.386 | 142.157 | **37.396** |

1. Total reading time

| **skill** | **Group** | **AIC_loess** | **AIC_lm** | **AIC_seglm** | **MSE_loess** | **MSE_lm** | **MSE_seglm** |
| --- | --- | --- | --- | --- | --- | --- | --- |
| Oral reading fluency | high | NA | 103577 | **103483** | 88.566 | 609.701 | **67.805** |
|  | low | NA | 112221 | **112153** | **153.718** | 750.613 | 171.96 |
| Verbal working memory | high | NA | 96924 | **96844** | **122.472** | 795.916 | 132.375 |
|  | low | NA | 119780 | **119718** | 106.846 | 515.087 | **88.735** |

1. First-pass regression

| **skill** | **Group** | **AIC_loess** | **AIC_lm** | **AIC_seglm** | **MSE_loess** | **MSE_lm** | **MSE_seglm** |
| --- | --- | --- | --- | --- | --- | --- | --- |
| Decoding | high | NA | 6270 | **6194** | **.00056** | .00234 | .00100 |
|  | low | NA | 7185 | **7170** | **.00043** | .00085 | .00050 |

**References**

Henderson, J. M., & Ferreira, F. (1990). Effects of foveal processing difficulty on the perceptual span in reading: implications for attention and eye movement control. *Journal of Experimental Psychology: Learning, Memory, and Cognition., 16*(3), 417-429.

Joseph, H. S. S. L., Nation, K., & Liversedge, S. P. (2013). Using Eye Movements to Investigate Word Frequency Effects in Children's Sentence Reading. *School Psychology Review, 42*(2), 207-222.

Kennison, S. M., & Clifton, C. (1995). Determinants of parafoveal preview benefit in high and low working memory capacity readers: implications for eye movement control. *Journal of Experimental Psychology: Learning, Memory, and Cognition, 21*(1), 68-81.

Kliegl, R., Nuthmann, A., & Engbert, R. (2006). Tracking the mind during reading: The influence of past, present and future words on fixation durations. *Journal of Experimental Psychology: General, 1*, 12-25.

Kuperman, V., Dambacher, M., Nuthmann, A., & Kliegl, R. (2010). The effect of word position on eye-movements in sentence and paragraph reading. *Quarterly Journal of Experimental Psychology, 63*(9), 1838-1857.

Kuperman, V., & Van Dyke, J. A. (2011). Effects of individual differences in verbal skills on eye-movement patterns during sentence reading. *Journal of Memory and Language, 65*(1), 42-73. doi:10.1016/j.jml.2011.03.002

Pynte, J., & Kennedy, A. (2006). An influence over eye movements in reading exerted from beyond the level of the word: Evidence from reading English and French. *Vision Research, 46*, 3786-3801.

Rayner, K. (1998). Eye movements in reading and information processing: 20 years of research. *Psychological Bulletin, 124*(3), 372-422.

Rayner, K., Cook, A. E., Juhasz, B. J., & Frazier, L. (2006). Immediate disambiguation of lexically ambiguous words during reading: evidence from eye movements. *British journal of psychology, 97*(4), 467-482. Retrieved from 10.1348/000712605X89363 [doi]

Rayner, K., & Duffy, S. A. (1986). Lexical complexity and fixation times in reading: effects of word frequency, verb complexity, and lexical ambiguity. *Memory and Cognition, 14*(3), 191-201.

Valle, A., Binder, K. S., Walsh, C. B., Nemier, C., & Bangs, K. E. (2013). Eye Movements, Prosody, and Word Frequency Among Average-and High-Skilled Second-Grade Readers. *School Psychology Review, 42*(2), 171-190.

Warren, T., White, S. J., & Reichle, E. D. (2009). Investigating the causes of wrap-up effects: Evidence from eye movements and E-Z Reader. *Cognition, 111*(1), 132-137. <http://dx.doi.org/10.1016/j.cognition.2008.12.011>
